# Multicellular Model Reveals the Mechanism of AEE Alleviating Vascular Endothelial Cell Injury via Anti-Inflammatory and Antioxidant Effects

**DOI:** 10.3390/ijms27020877

**Published:** 2026-01-15

**Authors:** Ji Feng, Qi Tao, Meng-Zhen Li, Zhi-Jie Zhang, Qin-Fang Yu, Jian-Yong Li

**Affiliations:** Key Lab of New Animal Drug of Gansu Province, Key Lab of Veterinary Pharmaceutical Development of Ministry of Agriculture and Rural Affairs, Lanzhou Institute of Husbandry and Pharmaceutical Sciences of CAAS, Lanzhou 730050, Chinalmz_678@163.com (M.-Z.L.);

**Keywords:** aspirin eugenol ester (AEE), vascular endothelial cells (VECs), metabolomics, oxidative stress, inflammation

## Abstract

Vascular endothelial injury is a key pathological characteristic of multiple diseases, such as atherosclerosis, stroke, and mastitis. Aspirin eugenol ester (AEE) has been confirmed to exert a significant protective effect on vascular endothelial injury. However, the universal action patterns and underlying mechanisms of AEE across different pathological scenarios have not been systematically elucidated. This study aimed to investigate the effect and mechanism of AEE in alleviating multiple vascular endothelial injury models. Nine vascular endothelial injury models were established by treating bovine aortic endothelial cells (BAECs), mouse aortic endothelial cells (MAECs), and human umbilical vein endothelial cells (Huvecs) with ethanol (EtOH), hydrogen peroxide (H_2_O_2_), and copper sulfate (CuSO_4_), respectively. The protective effects of AEE were systematically evaluated via morphological observation, detection of inflammatory responses, and oxidative stress markers. Furthermore, metabolomics was employed to identify and analyze differentially expressed metabolites between the nine model groups and AEE groups. AEE exerted protective effects on all nine vascular endothelial injury models, inhibiting inflammation and oxidative stress induced by all inducers. Metabolomic analysis revealed that the differentially expressed metabolites modulated by AEE in most models were primarily enriched in lipid metabolism, amino acid metabolism, coenzyme biosynthesis, and other related pathways. AEE could improve vascular endothelial injury by upregulating antioxidant substance which included eicosapentaenoic acid (EPA), choline, coenzyme A (CoA), glutathione (GSH), catalase (CAT) and superoxide dismutase (SOD), as well as downregulating substances that cause endothelial oxidative damage, including phytosphingosine (PS), palmitic acid (PA), and arachidonic acid (AA).

## 1. Introduction

Oxidative stress is defined as a state in which the production of reactive oxygen species (ROS) and other free radicals that are triggered by various endogenous and exogenous harmful stimuli exceeds the capacity of the antioxidant defense system to neutralize them, leading to the accumulation of these reactive species in the organism. Under normal physiological conditions, the redox reactions within cells are in a dynamic equilibrium. The antioxidant system of cells is composed of antioxidant enzymes such as SOD and CAT, as well as non-enzymatic antioxidants like GSH; the processes of the mitochondrial electron transport chain and oxidative reactions in the endoplasmic reticulum generate ROS and other free radicals, which can efficiently clean free radicals, thereby maintaining the stability of the intracellular environment [1,2].

The vascular endothelium serves as the first barrier and functional core of the vascular wall. As it directly contacts the blood, it is susceptible to the influence of chemical substances transported in the blood as well as physical factors. When the vascular endothelial cells (VECs) are stimulated by harmful factors such as hypertension, alcohol, heavy metal ion (such as copper and lead) poisoning, trauma, and inflammatory substances, they lead to mitochondrial damage in VECs, disrupting intracellular redox balance, which promotes the generation of intracellular reactive oxygen species (ROS) and simultaneously weakens the antioxidant defense system [2,3,4,5,6,7,8]. The combined effect of these two processes leads to excessive accumulation of ROS. Excess ROS impairs the structural and functional integrity of VECs. Additionally, ROS promote the upregulation of pro-inflammatory factors such as interleukin-6 (IL-6) and tumor necrosis factor alpha (TNF-α) and increase the release of adhesion molecules such as intercellular adhesion molecule-1(ICAM-1) and vascular cell adhesion molecule-1(VCAM-1). These changes in related factors further trigger a series of pathological reactions in VECs [9].

For humans, vascular-related oxidative stress plays a crucial role in the development and progression of cardiovascular diseases. ROS mediate the oxidation of low-density lipoprotein (LDL) to ox-LDL, which is then taken up by VECs and macrophages, forming foam cells that accumulate in the intima and initiate atherosclerosis. As plaques grow and intraplaque hemorrhaging occurs, it can ultimately lead to fatal events, such as myocardial infarction and stroke [10,11,12,13]. Additionally, long-term oxidative stress contributes to peripheral vascular disease, which affects limb circulation, in severe cases, leading to ulcers and gangrene [14,15,16]. In other animals, such as cattle, vascular endothelial oxidative stress also causes functional disorders and specific diseases. Due to hormonal fluctuations and nutritional stress, periparturient cows are prone to developing an oxidative stress microenvironment and a burst of ROS production in VECs [17,18,19]. This damages placental blood vessels, resulting in an inadequate blood supply to the fetus and reproductive disorders such as abortion and weak calves. Additionally, oxidative stress disrupts the microvasculature of the mammary glands, which interferes with milk secretion and transport. This reduces milk yield and increases the risk of mastitis [20,21]. Oxidative stress in beef cattle leads to peripheral vascular dysfunction, reduced blood flow to muscle tissues, and decreased growth efficiency. Furthermore, the damage to VECs can impair immunity, increasing the risk of respiratory and gastrointestinal infections. These infections directly result in economic losses in livestock production of beef cattle. Therefore, developing a deeper understanding of how oxidative stress contributes to vascular injury and identifying effective antioxidant interventions are significant for ensuring animal health [22,23,24].

AEE is a novel ester compound that is synthesized through a dehydration condensation reaction involving the carboxylic acid group of aspirin and the phenolic hydroxyl group of eugenol. Pharmacological studies demonstrate that AEE has multiple pharmacological activities, including anti-inflammatory and antioxidant effects. Previous research has confirmed that AEE exerts favorable protective effects on vascular endothelial injury models induced by various stimuli such as H_2_O_2_ or paraquat, with its mechanisms of action all involving the regulation of the antioxidant stress process [25,26,27]. However, existing studies on AEE for alleviating vascular endothelial injury and oxidative stress are primarily conducted in single models, which limits the ability to reveal its universal action patterns in complex pathological conditions. Additionally, there is a lack of systematic comparison and integration in the protective mechanisms of AEE. In-depth investigation of the common networks and specific nodes governing AEE-mediated protection against vascular endothelial injury induced by diverse factors will expand its specific application scenarios in related fields.

Metabolomics is a key component of systems biology. It focuses on the comprehensive qualitative and quantitative analysis of small-molecule metabolites within biological systems. The fundamental principle of metabolomics is to uncover correlation networks between metabolites and physiological or pathological states using high-sensitivity analytical techniques. Compared with other omics technologies, metabolomics has unique advantages that include directly reflecting the functional state of an organism, having a broad dynamic range of detection windows, and experiencing minimal interference from the genetic background. This enables metabolomics to directly capture the final effects induced by various stimuli in organisms [28,29]. Metabolomics has been used to expand the study of the pharmacological mechanisms of AEE. For instance, in the thrombosis model, AEE was found to have antithrombotic effects by regulating the phenylalanine-tyrosine–tryptophan and glutathione metabolism pathways. In the lipopolysaccharid-induced acute lung injury model, metabolomics analysis revealed that the levels of metabolites were significantly altered between the model and AEE groups. The metabolites were primarily enriched in key pathways, such as glutathione metabolism, cysteine metabolism, methionine metabolism, and glutamate metabolism [30,31]. These studies demonstrate that AEE can affect changes in metabolites related to oxidative stress pathways at the metabolic level. However, whether AEE improves vascular endothelial injury and oxidative stress status by regulating similar metabolites and their network pathways remains unclear. Therefore, this study employed chemical stimuli easily encountered in life activities—H_2_O_2_, EtOH, and CuSO_4_—to induce vascular endothelial injury models. The aim was to investigate the effects of AEE on changes in cellular metabolites and systematically clarify the dynamic characteristics of metabolites regulated by AEE intervention and their associated network pathways under the pathological conditions of vascular endothelial injury and oxidative stress. This clarification can not only provide key metabolic evidence for in-depth analysis of AEE’s mechanism in alleviating vascular endothelial injury and oxidative stress, but also lay a theoretical foundation for its clinical application in diseases related to vascular endothelial injury.

## 2. Results

### 2.1. Effects of EtOH, H_2_O_2_, and CuSO_4_ on Cell Viability in BAECs, MAECs, and Huvecs

**Figure 1:**
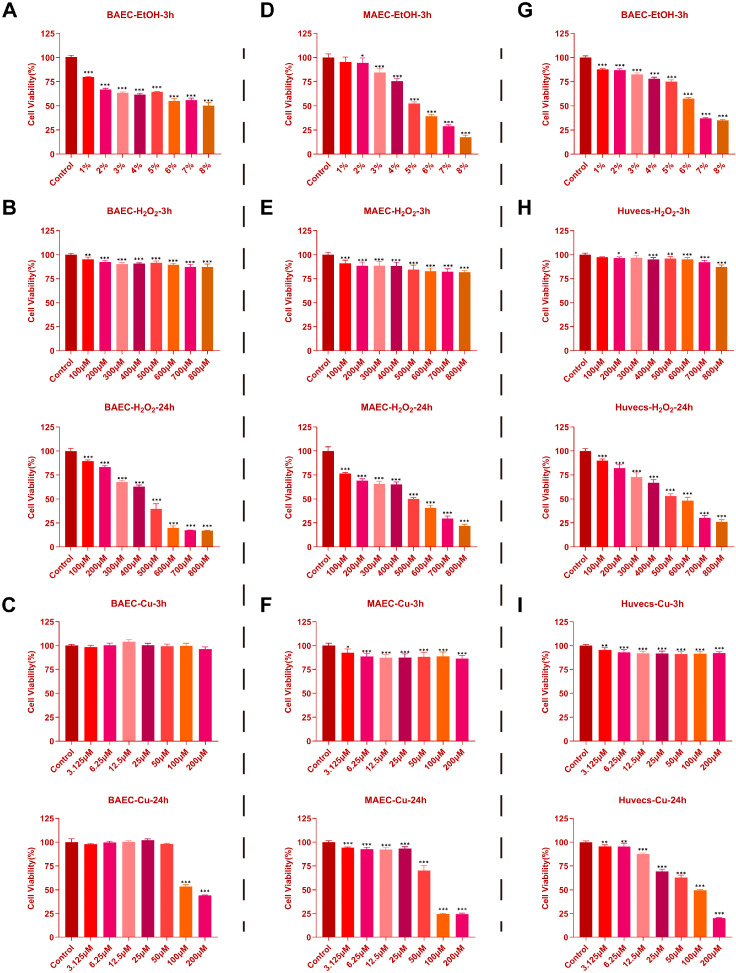
Effects of EtOH, H_2_O_2_, and CuSO_4_ on Cell Viability in BAECs, MAECs, and Huvecs. (**A**–**C**) Cell viability of BAECs treated with different concentrations of EtOH, H_2_O_2_ or CuSO_4_ (*n* = 6). (**D**–**F**) Cell viability of MAECs treated with different concentrations of EtOH, H_2_O_2_ or CuSO_4_ (*n* = 6). (**G**–**I**) Cell viability of Huvecs treated with different concentrations of EtOH, H_2_O_2_ or CuSO_4_ (*n* = 6). Data are represented as mean ± SD. Comparison among groups: * *p* < 0.05, ** *p* < 0.01, *** *p* < 0.001.

As shown in Figure 1A–C, EtOH treatment at 8% for 3 h reduced BAEC viability to 50.25%; H_2_O_2_ treatment for 24 h reduced BAEC viability to 39.70%; and CuSO_4_ treatment for 24 h reduced BAEC viability to 53.22%. From Figure 1D–F, EtOH treatment at 8% for 3 h reduced MAEC viability to 52.37%; H_2_O_2_ treatment for 24 h reduced MAEC viability to 50.2%; and CuSO_4_ treatment for 24 h reduced MAEC viability to 70.09%. EtOH treatment at 8% for 3 h reduced Huvecs viability to 57.36%; H_2_O_2_ treatment for 24 h reduced Huvecs viability to 53.17%; and CuSO_4_ treatment for 24 h reduced Huvecs viability to 49.33% (Figure 1G–I). Based on the literature [8,32,33], corresponding treatment concentrations of EtOH, H_2_O_2_, and CuSO_4_ that reduced cell viability to approximately 50% were selected for subsequent experiments.

### 2.2. Protective Effects of AEE Against EtOH, H_2_O_2_, and CuSO_4_ Induced Injury in BAECs, MAECs, and Huvecs

**Figure 2:**
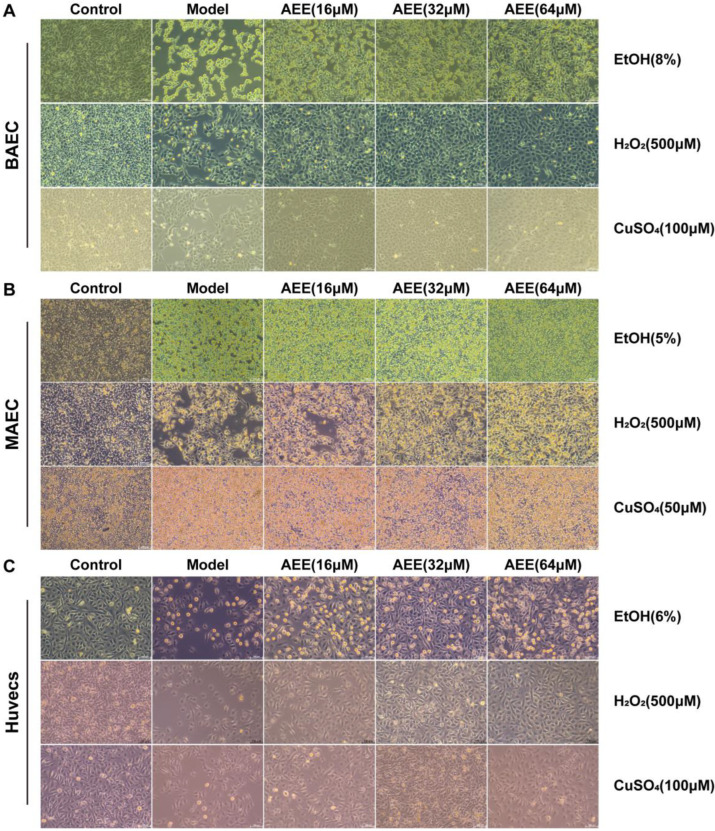
Effects of AEE on vascular endothelial injury models. (**A**) Effects of AEE pretreatment on EtOH-, H_2_O_2-_ or CuSO_4_-induced BAECs injury under 10× light microscopy, (Scale bar = 100 μm). (**B**) Effects of AEE pretreatment on EtOH-, H_2_O_2-_ or CuSO_4_-induced MAECs injury under 10× light microscopy, (Scale bar = 100 μm). (**C**) Effects of AEE and pretreatment on EtOH-, H_2_O_2-_ or CuSO_4_-induced Huvecs injury under 10× light microscopy, (Scale bar = 100 μm).

From Figure 2A and Figure S1A–C, compared with the control group, EtOH caused extensive detachment of BAECs and cell shrinkage. H_2_O_2_ and CuSO_4_ induced similar damage to BAECs, characterized by cell swelling, nuclear fragmentation, blurred cell boundaries, leakage of cytoplasmic content, and cell detachment. Pretreatment with AEE at 16, 32, or 64 μM exerted protective effects against damage induced by various initiators in BAECs. Microscopic examination and cell count results revealed that pretreatment with 64 μM AEE provided the optimal protection against EtOH-induced cell damage, while 32 μM AEE pretreatment was most effective against H_2_O_2_- and CuSO_4_-induced cell damage.

As shown in Figure 2B and Figure S1D–F, MAECs damage induced by EtOH and CuSO_4_ were similar, with cells exhibiting small-scale detachment and sporadic swelling; H_2_O_2_ induced marked pathological morphological changes in MAECs, including extensive cell death and detachment, cell fragmentation, and cytoplasmic content leakage. Pretreatment with AEE at 16, 32, or 64 μM exerted protective effects against damage induced by various initiators in MAECs. Microscopic examination and cell count results revealed that pretreatment with 64 μM AEE provided the optimal protection against EtOH- and H_2_O_2_-induced cell damage, whereas 32 μM AEE pretreatment was most effective against CuSO_4_-induced cell damage.

Compared with the control group, EtOH, H_2_O_2_, and CuSO_4_ induced significant morphological changes in Huvecs, including partial cell swelling, cell membrane breakdown, indistinct cell boundaries, and extensive cell detachment. Pretreatment with AEE at 16, 32, or 64 μM exerted protective effects against damage induced by various inducers in Huvecs. Microscopic observation and cell count results showed that pretreatment with 32 μM AEE provided the optimal protection against EtOH-, H_2_O_2_-, and CuSO_4_-induced cell damage (Figure 2C and Figure S1G–I).

### 2.3. AEE Alleviates Inflammation and Oxidative Stress in Various VECs

#### 2.3.1. AEE Alleviates EtOH-, H_2_O_2_-, or CuSO_4_-Induced Inflammation and Oxidative Stress in BAECs

**Figure 3:**
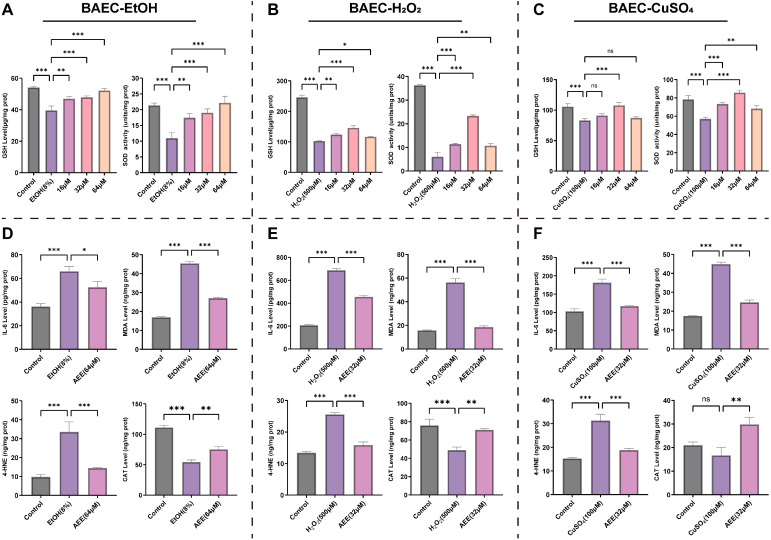
AEE alleviates inflammation and oxidative stress in BAECs. (**A**–**C**) The intracellular GSH levels and SOD activity of BAECs in each group (*n* = 3). (**D**–**F**) The intracellular IL-6, MDA, 4-HNE and CAT level of BAECs in each group (*n* = 3). The following experiments all include three groups, (**D**): Control, EtOH (8%), AEE (64 μM); (**E**): Control, H_2_O_2_ (500 μM), AEE (32 μM); (**F**): Control, CuSO_4_ (100 μM), AEE (32 μM). Data are represented as mean ± SD. Comparison among groups: ns *p* > 0.05, * *p* < 0.05, ** *p* < 0.01, *** *p* < 0.001.

As shown in Figure 3A–C, compared with the control group, EtOH, H_2_O_2_, and CuSO_4_ all significantly reduced intracellular GSH levels and SOD activity in BAECs. Pretreatment with AEE at various concentrations improved these changes to different extents. For the EtOH-induced BAEC injury model, 64 μM AEE exhibited the mostly notable ameliorative effects on GSH levels and SOD activity. For the H_2_O_2_- or CuSO_4_-induced models, 32 μM AEE showed the most significant improvements in both parameters. Based on morphological observations and the above-mentioned indicators, 32 μM AEE was selected for the H_2_O_2_- or CuSO_4_-induced model, and 64 μM AEE for the EtOH-induced model as the subsequent treatment concentration.

Compared with the control group, EtOH, H_2_O_2_, and CuSO_4_ all significantly increased IL-6, MDA, and 4-HNE levels in BAECs. EtOH and H_2_O_2_ significantly decreased CAT levels in BAECs, while CuSO_4_ had no significant effect on CAT levels but showed a trend toward reduction. Pretreatment with AEE at corresponding concentrations significantly inhibited the increases in IL-6, MDA, and 4-HNE levels and elevated CAT levels in BAECs across all inducer–induced injury models (Figure 3D–F).

#### 2.3.2. AEE Alleviates EtOH-, H_2_O_2_-, or CuSO_4_-Induced Inflammation and Oxidative Stress in MAECs

**Figure 4:**
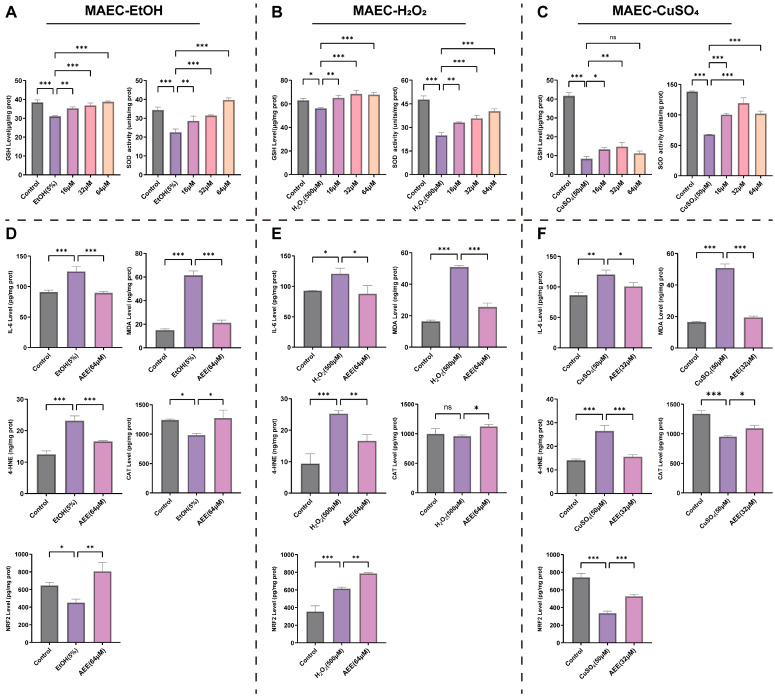
AEE alleviates inflammation and oxidative stress in MAECs. (**A**–**C**) The intracellular GSH levels and SOD activity of MAECs in each group (*n* = 3). (**D**–**F**) The intracellular IL-6, MDA, 4-HNE, CAT and NRF2 level of MAECs in each group (*n* = 3). The following experiments all include three groups, (**D**): Control, EtOH (5%), AEE (64 μM); (**E**): Control, H_2_O_2_ (500 μM), AEE (64 μM); (**F**): Control, CuSO_4_ (50 μM), AEE (32 μM). Data are represented as mean ± SD. Comparison among groups: ns *p* > 0.05, * *p* < 0.05, ** *p* < 0.01, *** *p* < 0.001.

As shown in Figure 4A–C, compared with the control group, EtOH, H_2_O_2_, and CuSO_4_ all significantly reduced intracellular GSH levels and SOD activity in MAECs. AEE at various concentrations alleviated these reductions. For the EtOH-induced MAEC injury model, 64 μM AEE exhibited the mostly notable alleviation of GSH levels and SOD activity. For the H_2_O_2_-induced model, both 32 μM and 64 μM AEE significantly increased GSH level with similar efficacy, while 64 μM AEE showed the most significant elevation in SOD activity. For the CuSO_4_-induced model, 32 μM AEE demonstrated the most significant alleviative effects on GSH levels and SOD activity. Based on morphological observations and above-mentioned indicators, 64 μM AEE was selected for the H_2_O_2_- or EtOH-induced model, and 32 μM AEE for the CuSO_4_ -induced model as the subsequent treatment concentration.

Compared with the control group, EtOH and CuSO_4_ both significantly increased IL-6, MDA, and 4-HNE levels in MAECs and decreased NRF2 and CAT levels. H_2_O_2_ significantly increased IL-6, MDA, 4-HNE, and NRF2 levels in MAECs without affecting CAT levels. Pretreatment with AEE at corresponding concentrations significantly reduced IL-6, MDA, and 4-HNE levels and elevated NRF2 and CAT levels in MAECs across all inducer–induced injury models (Figure 4D–F).

#### 2.3.3. AEE Alleviates EtOH-, H_2_O_2_-, or CuSO_4_-Induced Inflammation and Oxidative Stress in Huvecs

**Figure 5:**
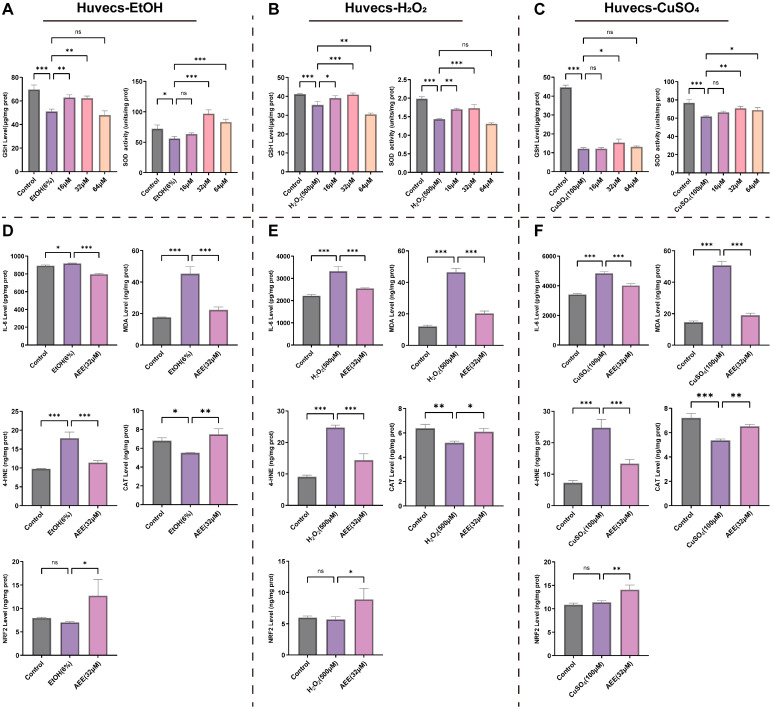
AEE alleviates inflammation and oxidative stress in Huvecs. (**A**–**C**) The intracellular GSH levels and SOD activity of Huvecs in each group (*n* = 3). (**D**–**F**) The intracellular IL-6, MDA, 4-HNE, CAT and NRF2 level of Huvecs in each group (*n* = 3). The following experiments all include three groups, (**D**): Control, EtOH (6%), AEE (32 μM); (**E**): Control, H_2_O_2_ (500 μM), AEE (32 μM); (**F**): Control, CuSO_4_ (100 μM), AEE (32 μM). Data are represented as mean ± SD. Comparison among groups: ns *p* > 0.05, * *p* < 0.05, ** *p* < 0.01, *** *p* < 0.001.

As shown in Figure 5A–C, compared with the control group, EtOH, H_2_O_2_, and CuSO_4_ all significantly reduced intracellular GSH levels and SOD activity in Huvecs. AEE at various concentrations alleviated these changes in GSH levels and SOD activity induced by each inducer. For the EtOH-, H_2_O_2_-, and CuSO_4_-induced Huvecs injury models, 32 μM AEE exhibited the most pronounced alleviation of GSH levels and SOD activity. Based on its performance in mitigating cell injury and modulating GSH levels and SOD activity across all models, 32 μM AEE was selected as the concentration for subsequent experiments.

Compared with the control group, EtOH, H_2_O_2_, and CuSO_4_ all significantly increased IL-6, MDA, and 4-HNE levels in Huvecs and decreased CAT levels, while having no significant effect on NRF2 levels. Pretreatment with 32 μM AEE significantly reduced IL-6, MDA, and 4-HNE levels and elevated NRF2 and CAT levels in Huvecs across all injury models (Figure 5D–F).

### 2.4. Metabolomic Analysis

#### 2.4.1. Metabolomic Analysis of the EtOH-Induced Cell Injury Model

**Figure 6:**
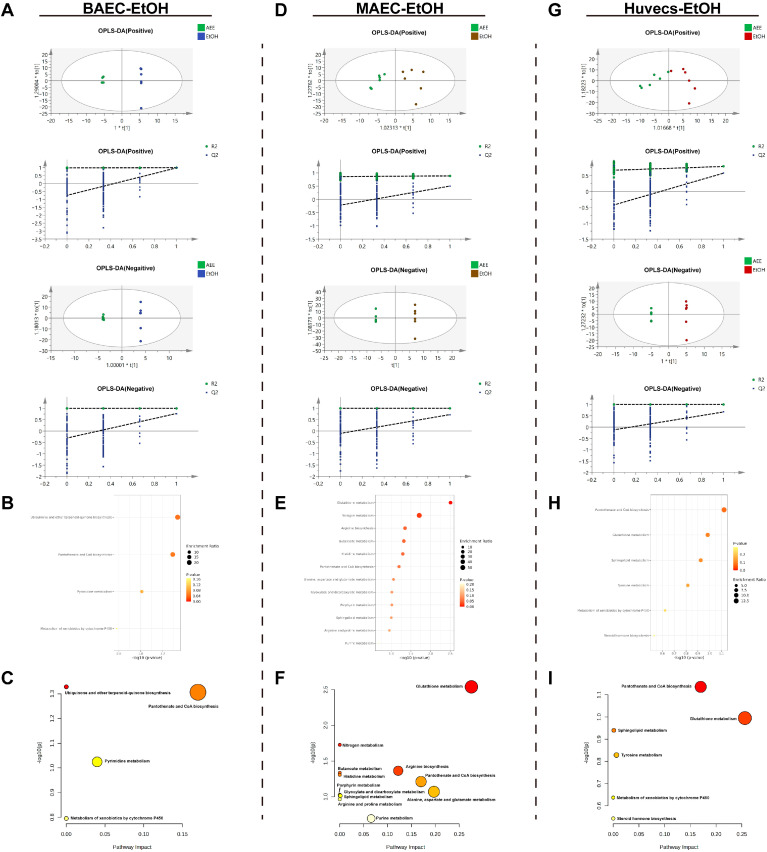
EtOH-induced cell injury model metabolomics analysis. (**A**–**C**) EtOH, (**A**): ESI+: R^2^Y = 1.000, Q^2^ = 0.978, ESI−: R^2^Y = 1.000, Q^2^ = 0.770. (**B**): BAECs metabolite enrichment analysis. (**C**): BAECs metabolite pathway analysis. (**D**–**F**) EtOH, (**D**): ESI+: R^2^Y = 0.885, Q^2^ = 0.501, ESI−: R^2^Y = 1.000, Q^2^ = 0.720. (**E**): MAECs metabolite enrichment analysis. (**F**): MAECs metabolite pathway analysis. (**G**–**I**) EtOH, (**G**): ESI+: R^2^Y = 0.784, Q^2^ = 0.573, ESI−: R^2^Y = 1.000, Q^2^ = 0.665. (**H**): Huvecs metabolite enrichment analysis. (**I**): Huvecs metabolite pathway analysis.

As shown in Figure 6A–C, OPLS-DA analysis was performed on the metabolite data of the AEE group versus the model group in the EtOH-induced BAEC injury model. The results indicated that the model exhibited no overfitting and had good predictive capacity. After conditional screening, four differentially expressed metabolites (DEMs) were identified between the AEE and model groups: AEE treatment significantly upregulated three DEMs and downregulated one DEM (Table 1). The DEMs were primarily enriched in pathways, *Ubiquinone and other terpenoid–quinone biosynthesis*, *Pantothenate and CoA biosynthesis*, *Pyrimidine metabolism*, and *Metabolism of xenobiotics by cytochrome P450*.

From Figure 6D–F, OPLS-DA analysis was performed on the metabolite data of the AEE group versus the model group in the EtOH-induced MAEC injury model. The results indicated that the model exhibited no overfitting and had good predictive capacity. After conditional screening, five DEMs were identified between the AEE and model groups: AEE treatment significantly upregulated four DEMs and downregulated one DEM (Table 1). The DEMs were primarily enriched in pathways *Glutathione metabolism*, *Nitrogen metabolism*, *Arginine biosynthesis*, *Butanoate metabolism*, *Histidine metabolism*, *Pantothenate and CoA biosynthesis*, *Alanine, aspartate and glutamate metabolism*, *Glyoxylate and dicarboxylate metabolism*, *Porphyrin metabolism*, *Sphingolipid metabolism*, *Arginine and proline metabolism*, and *Purine metabolism*.

In the EtOH-induced Huvecs injury model, OPLS-DA analysis of the AEE and model group metabolite data showed no overfitting and good predictive performance. Conditional screening identified six DEMs: AEE treatment significantly upregulated all six DEMs (Table 1). The DEMs were primarily enriched in pathways including *Pantothenate and CoA biosynthesis*, *Glutathione metabolism*, *Sphingolipid metabolism*, *Tyrosine metabolism*, *Metabolism of xenobiotics by cytochrome P450*, and *Steroid hormone biosynthesis* (Figure 6G–I).

#### 2.4.2. Metabolomic Analysis of the H_2_O_2_-Induced Cell Injury Model

**Figure 7:**
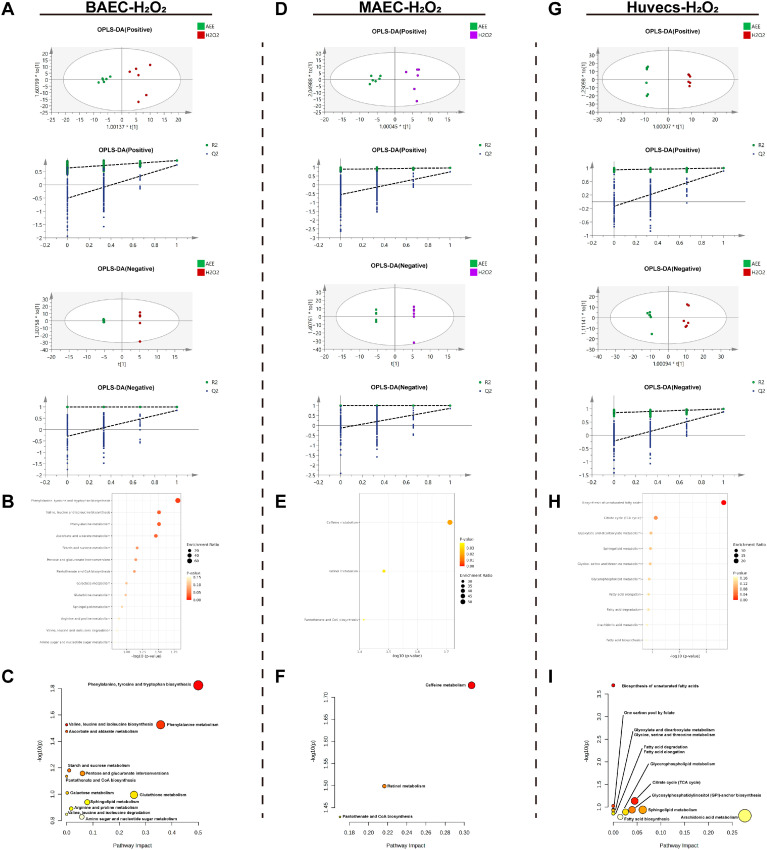
H_2_O_2_-induced cell injury model metabolomics analysis. (**A**–**C**) H_2_O_2_, (**A**): ESI+: R^2^Y = 0.916, Q^2^ = 0.747, ESI−: R^2^Y = 1.000, Q^2^ = 0.847. (**B**): BAECs metabolite enrichment analysis. (**C**): BAECs metabolite pathway analysis. (**D**–**F**) H_2_O_2_, (**D**): ESI+: R^2^Y = 0.959, Q^2^ = 0.726, ESI−: R^2^Y = 0.986, Q^2^ = 0.857. (**E**): MAECs metabolite enrichment analysis. (**F**): MAECs metabolite pathway analysis. (**G**–**I)** H_2_O_2_, (**G**): ESI+: R^2^Y = 0.999, Q^2^ = 0.915, ESI−: R^2^Y = 0.994, Q^2^ = 0.880. (**H**): Huvecs metabolite enrichment analysis. (**I**): Huvecs metabolite pathway analysis.

As shown in Figure 7A–C, OPLS-DA analysis was performed on the metabolite data of the AEE group versus the model group in the H_2_O_2_-induced BAEC injury model. The results indicated that the model exhibited no overfitting and had good predictive capacity. After conditional screening, six DEMs were identified between the AEE and model groups: all six DEMs were significantly upregulated by AEE treatment (Table 2). These DEMs were primarily enriched in pathways including *Phenylalanine*, *tyrosine and tryptophan biosynthesis*, *Valine*, *leucine and isoleucine biosynthesis*, *Phenylalanine metabolism*, *Ascorbate and aldarate metabolism*, *Starch and sucrose metabolism*, *Pentose and glucuronate interconversions*, *Pantothenate and CoA biosynthesis*, *Galactose metabolism*, *Glutathione metabolism*, *Sphingolipid metabolism*, *Arginine and proline metabolism*, *Valine*, *leucine and isoleucine degradation*, and *Amino sugar and nucleotide sugar metabolism*.

From Figure 7D–F, OPLS-DA analysis of the metabolite data from the AEE and model groups in the H_2_O_2_-induced MAEC injury model revealed no overfitting and good predictive capacity. Conditional screening identified three DEMs: three DEMs were significantly downregulated by AEE treatment (Table 2). These DEMs were primarily enriched in pathways including *Caffeine metabolism*, *Retinol metabolism*, and *Pantothenate and CoA biosynthesis*.

In the H_2_O_2_-induced Huvecs injury model, OPLS-DA analysis was performed on the metabolite data of the AEE group versus the model group. The results indicated that the model exhibited no overfitting and had good predictive capacity. After conditional screening, six DEMs were identified between the AEE and model groups: AEE treatment significantly upregulated three DEMs and downregulated three DEMs (Table 2). These DEMs were primarily enriched in pathways including *Biosynthesis of unsaturated fatty acids*, *One carbon pool by folate*, *Citrate cycle (TCA cycle)*, *Glyoxylate and dicarboxylate metabolism*, *Sphingolipid metabolism*, *Glycine*, *serine and threonine metabolism*, *Glycosylphosphatidylinositol (GPI)-anchor biosynthesis*, *Glycerophospholipid metabolism*, *Fatty acid elongation*, *Fatty acid degradation*, *Arachidonic acid metabolism*, and *Fatty acid biosynthesis* (Figure 7G–I).

#### 2.4.3. Metabolomic Analysis of the CuSO_4_-Induced Cell Injury Model

**Figure 8:**
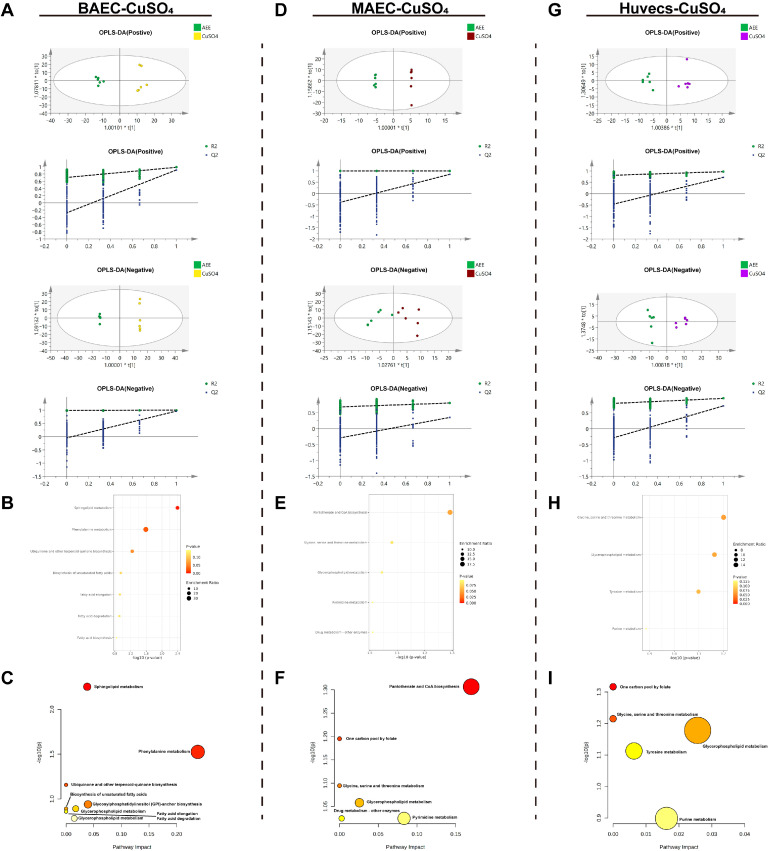
CuSO_4_-induced cell injury model metabolomics analysis. (**A**–**C**) CuSO_4_, (**A**): ESI+: R^2^Y = 0.984, Q^2^ = 0.909, ESI−: R^2^Y = 1.000, Q^2^ = 0.963. (**B**): BAECs metabolite enrichment analysis. (**C**): BAECs metabolite pathway analysis. (**D**–**F**) CuSO_4_, (**D**): ESI+: R^2^Y = 1, Q^2^ = 0.844, ESI−: R^2^Y = 0.804, Q^2^ = 0.351. (**E**): MAECs metabolite enrichment analysis. (**F**): MAECs metabolite pathway analysis. (**G**–**I**) CuSO_4_, (**G**): ESI+: R^2^Y = 0.968, Q^2^ = 0.715, ESI−: R^2^Y = 0.953, Q^2^ = 0.715. (**H**): Huvecs metabolite enrichment analysis. (**I**): Huvecs metabolite pathway analysis.

As shown in Figure 8A–C, OPLS-DA analysis was performed on the metabolite data of the AEE group versus the model group in the CuSO_4_-induced BAEC injury model. The results indicated that the model exhibited no overfitting and had good predictive capacity. After conditional screening, six differentially expressed metabolites (DEMs) were identified between the AEE and model groups: AEE treatment significantly upregulated two DEMs and downregulated four DEMs (Table 3). The DEMs were primarily enriched in pathways, *Sphingolipid metabolism*, *Phenylalanine metabolism*, *Ubiquinone and other terpenoid–quinone biosynthesis*, *Biosynthesis of unsaturated fatty acids*, *Glycosylphosphatidylinositol (GPI)-anchor biosynthesis*, *Glycerophospholipid metabolism*, *Fatty acid elongation*, *Fatty acid degradation*, and *Fatty acid biosynthesis*.

From Figure 8D–F, OPLS-DA analysis was performed on the metabolite data of the AEE group versus the model group in the CuSO_4_-induced MAEC injury model. The results indicated that the model exhibited no overfitting and had good predictive capacity. After conditional screening, four differentially expressed metabolites (DEMs) were identified between the AEE and model groups: AEE treatment significantly upregulated one DEM and downregulated three DEMs (Table 3). The DEMs were primarily enriched in pathways, *Pantothenate and CoA biosynthesis*, *One carbon pool by folate*, *Glycine*, *serine and threonine metabolism*, *Glycerophospholipid metabolism*, *Drug metabolism—other enzymes*, and *Pyrimidine metabolism*.

In the CuSO_4_-induced Huvecs injury model, OPLS-DA analysis of the AEE and model group metabolite data showed no overfitting and good predictive performance. Conditional screening identified three DEMs: AEE treatment significantly upregulated two DEMs and downregulated one DEM (Table 3). The DEMs were primarily enriched in pathways including *One carbon pool by folate*, *Glycine*, *serine and threonine metabolism*, *Glycerophospholipid metabolism*, *Tyrosine metabolism*, and *Purine metabolism* (Figure 8G–I).

## 3. Discussion

This study used EtOH, H_2_O_2_, and CuSO_4_ to establish nine vascular endothelial injury models for BAECs, MAECs, and Huvecs. AEE was used as a pretreatment, and the best concentration of AEE was determined based on the levels of inflammation and oxidative stress-related factors for subsequent metabolomic analysis. The results showed that AEE significantly improved cell viability, reduced the release of inflammatory factors, and enhanced the activity of antioxidant enzymes in multiple cell models. Metabolomic results indicated that AEE primarily exerts its pharmacological effects by regulating intracellular lipid metabolism, amino acid metabolism, and coenzyme biosynthesis, targeting key metabolic nodes involved in oxidative stress and inflammatory responses, thereby effectively alleviating vascular endothelial damage.

VECs, as a monolayer barrier of the vascular wall, play a crucial role in maintaining the integrity and functional homeostasis of blood vessels. Numerous studies have confirmed that vascular endothelial injury is not only a key early event in the development of various cardiovascular diseases, such as atherosclerosis, thrombosis, and hypertension, but also a common pathological basis for the progression of these diseases [34,35,36]. After endothelial cell injury, the synthesis of vasodilatory and antithrombotic factors, such as nitric oxide and prostacyclin, decreases, while the expression of endothelial adhesion molecules and pro-inflammatory factors increases. This ultimately leads to vascular dysfunction, platelet activation, and leukocyte infiltration [37,38,39]. Notably, this damage is closely associated with oxidative–reductive imbalance. Under conditions such as physical injury, chemical stimulation, or pathogen infection, the excessive production of ROS (superoxide anion radicals (O_2_•^−^) and H_2_O_2_) and the impairment of antioxidant defense systems (such as GSH, SOD, and CAT) of the endothelial cells lead to excessive oxidative stress. Oxidative stress within VECs subsequently damages cell structures and functions through pathways such as lipid peroxidation, protein oxidation, and DNA damage [40,41,42]. Current research indicates that AEE has favorable pharmacological activity in protecting against VEC injury and alleviating oxidative stress. Conducting systematic metabolomic studies using multiple models to investigate metabolic changes can contribute to comprehensively elucidating the specific mechanisms of action of AEE. This can provide critical metabolic evidence for understanding the effects of AEE on improving VEC injury and oxidative stress, and can also lay a theoretical foundation for its clinical application in diseases related to VEC injury.

Sphingolipids, as core structural components of the cell membrane phospholipid bilayer, play crucial roles in regulating key biological processes such as cell proliferation, differentiation, development, apoptosis, angiogenesis, and immune responses [43]. PS, a characteristic component of the sphingolipid family, is widely distributed in both animals and plants and exhibits multiple pharmacological activities, including anti-inflammatory, antioxidant, antimicrobial, and antitumor [44,45,46]. Studies have shown that PS can effectively alleviate Staphylococcus aureus-induced mastitis by inhibiting the activation of pro-inflammatory signaling pathways such as NF-κB/NLRP3, reducing the release of inflammatory factors such as IL-1β and TNF-α. Additionally, PS can target and repair blood–milk barrier damage by upregulating the expression levels of tight junction proteins, ZO-1, Occludin, and Claudin-3, thereby restoring the integrity and permeability of the barrier [47]. Fatty acid metabolism is deeply involved in the regulation of intracellular oxidative stress. PA, a common saturated fatty acid, is primarily derived from exogenous dietary intake or endogenous synthesis [48]. PA can lead to oxidative stress and inflammatory cascades in VECs by inducing lipid peroxidation, releasing pro-inflammatory factors, and causing neutrophil apoptosis, that lead to impair endothelium-dependent vasodilation and disruption of barrier integrity [49,50]. Clinical studies have confirmed that elevated plasma PA levels are significantly associated with an increased risk of cardiovascular diseases such as atherosclerosis and coronary heart disease. This indicates that PA plays an important role in the development and progression of these diseases [51,52]. EPA is an ω-3 polyunsaturated fatty acid and a key regulator of cardiovascular health. EPA has been shown to improve various cardiovascular conditions, including atrial fibrillation, atherosclerosis, thrombosis, inflammation, and sudden cardiac death [53,54]. The mechanisms by which EPA mitigates stress-related vascular inflammation are multidimensional. Specifically, EPA reduces the release of inflammatory factors (IL-6, TNF-α) and vascular adhesion molecules (ICAM-1), inhibits leukocyte chemotaxis, regulates lipid metabolism (TG, LDL, and PA), and promotes angiogenesis. Collectively, these actions reduce endothelial cell lipid peroxidation and inflammatory damage, thereby maintaining vascular homeostasis [55,56,57,58,59]. AA is an essential component of cell membrane phospholipids that plays a vital role in numerous physiological and pathological processes, particularly the regulation of inflammation and cardiovascular function. When VECs are stimulated or activated, AA is released from membrane phospholipids by phospholipase A2 and is subsequently metabolized by enzymes such as cyclooxygenase and lipoxygenase to produce inflammatory mediators, including thromboxane A2, prostaglandins, and leukotrienes. Abnormal elevation in these pro-inflammatory metabolites can lead to acute and chronic inflammation, exacerbate vascular inflammation, and contribute to the development of diseases such as atherosclerosis, thrombosis, asthma, and renal inflammation [60,61,62,63]. Choline is an essential nutrient and a key component of the phosphatidylcholine in cell membranes, as well as a precursor for acetylcholine. Choline is crucial for maintaining cellular homeostasis, and its deficiency can lead to multi-organ inflammatory responses, mitochondrial dysfunction, and abnormally high levels of oxidative stress, resulting in organ damage to the brain and kidneys and the development of a fatty liver [64,65,66,67,68]. Choline has a clear antagonistic effect against certain heavy metal toxicities, effectively preventing lead-induced HepG2 damage and significantly inhibiting lipid peroxidation, highlighting its important protective role against oxidative stress damage [69]. When cells are exposed to various stimuli, the oxidative stress in cell is induced. Subsequently, excessive ROS attack the polyunsaturated fatty acids in the cell membrane, which leads to the generation of lipid peroxidation products, MDA and 4-HNE. These compounds directly participate in the vascular inflammation and functional disorders, the pathological process of VECs, by further attacking the cell membrane, inducing cell death and inflammatory responses [70,71]. Therefore, elevated levels of MDA and 4-HNE reflect the degree of lipid peroxidation damage to VECs, indicating damage to the integrity and function of the VECs [72,73,74,75].

In this study, VEC injury models were established using EtOH, H_2_O_2_, or CuSO_4_. Oxidative stress and inflammation-related indicators were systematically detected. The levels of the inflammatory factor IL-6 and the oxidative stress markers MDA and 4-HNE were significantly higher in the different model groups than in the normal control group. This indicated that oxidative stress and activated inflammatory responses in VECs are triggered by EtOH, H_2_O_2_, and CuSO_4_. Following pretreatment with AEE, the abnormal elevation in these indicators was significantly inhibited. This suggests that AEE alleviated oxidative stress injury in VECs induced by different stimuli. The types of differential metabolites between the AEE group and model group varied across different VEC injury models. In the three VEC injury models induced by EtOH, 4′-phosphopantothenoylcysteine (4′-PPC) was a common differential metabolite. Among them, PS and GSH were common differential metabolites in the MAEC and Huvecs injury models. In the MAEC and Huvecs injury models induced by CuSO_4_, choline was a common differential metabolite. Although no common differential metabolites were found among the three VEC injury models induced by H_2_O_2_, it was observed that AEE exerted significant regulatory effects on lipid metabolism-related metabolites, such as PA, AA, EPA, choline, and sphinganine, and amino acid metabolism-related metabolites, such as GSH, proline, and phenylalanine, in different H_2_O_2_-induced VEC injury models. Overall, although differential metabolites may vary within models induced by the same agent or in the same cell type, those repeatedly identified across different VEC injury models—such as 4′-PPC, PS, choline, PA, and GSH—are likely key regulatory metabolites through which AEE improves VEC injury and oxidative stress. Further metabolomic enrichment analysis revealed that in nine VEC injury models, differential metabolites between the AEE group and the model group were mainly enriched in multiple lipid metabolic pathways, including *Sphingolipid metabolism*, *Fatty acid elongation*, *Fatty acid degradation*, *Fatty acid biosynthesis*, *Biosynthesis of unsaturated fatty acid*, *Glycerophospholipid metabolism*, and *Arachidonic acid metabolism*. This suggests that AEE may protect the endothelium by regulating the lipid metabolic network. Specifically, AEE pretreatment led to an upregulation of PS, choline, and EPA levels, while the levels of PA and AA were reduced in the VECs injury model. It is speculated that PS might enhance barrier function by maintaining membrane stability, that choline might reduce damage by inhibiting lipid peroxidation, and that EPA might exert protective effects by regulating inflammatory factors and lipid metabolism. The reduction in PA and AA levels may decrease the production of pro-inflammatory and pro-oxidative stress mediators. These findings indicated that AEE improves vascular endothelial injury by bidirectionally regulating lipid metabolic balance in two ways: enhanced cellular antioxidant and anti-inflammatory capabilities via increasing the levels of PS, EPA, and choline, and reducing the accumulation of PA and AA, thereby lowering the oxidative stress cascade and inflammatory amplification effects.

GSH is an important non-enzymatic antioxidant in the organism, composed of glutamic acid, cysteine, and glycine. It protects cells by directly reducing ROS, thereby preventing oxidative damage. GSH is also a key indicator of cellular redox status. Under conditions that induce vascular oxidative stress, the excessive production of ROS can deplete GSH, thereby weakening the cell’s antioxidant capacity and exacerbating oxidative damage, lipid peroxidation, and functional impairment in VECs [76,77,78,79,80,81]. SOD and CAT are the primary enzymatic antioxidant defense systems in VECs. SOD catalyzes the dismutation of O_2_•^−^ into H_2_O_2_, and CAT breaks down H_2_O_2_ into water and oxygen, effectively preventing ROS-mediated oxidative damage [82,83,84]. In vascular oxidative stress, the decline in enzyme activity or insufficient expression of SOD and CAT can lead to the accumulation of O_2_•^−^ and H_2_O_2_, further exacerbating oxidative damage and functional impairment in VECs. Various compounds have been shown to enhance vascular endothelial antioxidant levels by increasing the levels and activity of SOD and CAT [79,85,86,87]. NRF2 is a key transcription factor in cellular antioxidant responses. When VECs are damaged by oxidative stress, NRF2 translocate to the nucleus and binds to antioxidant response elements, thereby activating the expression of antioxidant enzymes such as SOD, CAT, NQO1, and HO-1. This enhances the antioxidant defense capacity of VECs, improving cell injury and functional disorders [88,89,90,91,92]. In this study, the levels of the non-enzymatic antioxidant GSH and key components of the enzymatic defense system, SOD and CAT, were significantly downregulated in the majority of the model groups. This is in line with the classic mechanism where excessive ROS consumption of antioxidants and reduction in antioxidant enzyme levels occur under oxidative stress, indicating impaired antioxidant capacity in VECs. As a core transcription factor in the antioxidant response, the expression of NRF2 was significantly downregulated in most models. However, in the H_2_O_2_-induced MAEC injury model, the expression of NRF2 was significantly upregulated. This may represent a compensatory feedback response to enhance the expression of antioxidant genes via the NRF2 pathway, thereby mitigating ROS-induced damage in MAECs [93,94]. Although some vascular endothelial injury models did not show significant changes in CAT and NRF2 levels, overall, the redox balance was disrupted, and the cellular antioxidant defense system was in a state of functional decline in the various model groups. Pretreatment with AEE consistently demonstrated an antioxidant repair effect across different VECs damage models induced by various inducers. AEE significantly upregulated the levels of GSH, SOD, and CAT, thereby directly enhancing the ability of VECs to eliminate ROS. Further metabolomic analysis revealed that the differential metabolites between the AEE group and the model group were primarily enriched in *Glutathione metabolism*, *Phenylalanine metabolism*, and *Arginine and proline metabolism*. GSH was significantly upregulated in different AEE-treated vascular endothelial injury models, consistent with the initial measurements. Additionally, in some models, glutamic acid, proline, and phenylalanine were also significantly upregulated. Glutamic acid, as a precursor for GSH synthesis, facilitates the production of GSH, suggesting that AEE may enhance endogenous GSH synthesis by increasing the supply of GSH precursors. Proline and phenylalanine have been reported to upregulate the expression of antioxidant genes, such as NRF2, thereby exerting an antioxidant effect [95,96,97]. AEE enhanced the antioxidant capacity of VECs primarily through both promoting the synthesis of antioxidant substances (GSH) and increasing the level of the enzymatic defense system (SOD, CAT). Additionally, AEE may regulate amino acid metabolism to reinforce long-term homeostasis of the antioxidant defense at the transcriptional level, thereby alleviating oxidative stress injury of VECs.

CoA is a key cofactor in biological systems, widely involved in biochemical reactions such as acetylation and carboxylation. It plays a crucial role in energy metabolism, lipid metabolism, and cell proliferation signaling. Studies have shown that the supplementation of acetyl-CoA can alleviate H_2_O_2_-induced senescence in VECs. Additionally, Stearoyl-CoA Desaturase 1, which is a CoA-dependent enzyme, can reduce the elevated expression of IL-6 and IL-8 induced by PA overload, and reduce endoplasmic reticulum stress, thereby mitigating VEC death and improving the progression of atherosclerosis [98,99,100]. Notably, 4′-PPC is a key intermediate in the biosynthesis of CoA. Therefore, the levels of 4′-PPC can dynamically reflect the activity of the CoA biosynthesis pathway [101,102]. In this study, metabolic changes related to CoA metabolism were detected in endothelial injury models induced by EtOH, H_2_O_2_, and CuSO_4_. The results showed that, compared to the model groups, the levels of 4′-PPC were significantly upregulated in all model groups pretreated with AEE. Given the biological significance of 4′-PPC as an intermediate in CoA biosynthesis, this phenomenon suggests that AEE may activate the CoA biosynthesis pathway, thereby increasing intracellular CoA levels. Given the biological significance of 4′-PPC as an intermediate in CoA biosynthesis and the known functions of CoA, the phenomenon suggested that AEE might enhance the CoA biosynthesis pathway, thereby increasing intracellular CoA levels. Adequate CoA supply could enhance antioxidant-related metabolism, regulate lipid and energy metabolism, and directly eliminate excess ROS, thereby enhancing cellular antioxidant capacity and ultimately improving the state of endothelial injury. This study revealed that AEE exerts multidimensional regulation on lipid metabolism, amino acid metabolism, and coenzyme synthesis pathways. It identified multiple lipids, GSH, and CoA as key metabolites through which AEE enhanced antioxidant capacity and improved vascular endothelial injury. First, AEE reduced the levels of pro-inflammatory and pro-oxidative stress metabolites and inflammatory substances within VECs, thereby alleviating oxidative stress cascades and inflammatory amplification effects. Second, AEE upregulated the levels of antioxidants, EPA, PS, CoA, and GSH, and enhanced the activity of important antioxidant enzymes, SOD and CAT. These mechanisms work synergistically to ultimately improve oxidative stress, cell function, and alleviate damage in VECs.

Cross-validation using multiple endothelial injury models preliminarily identified the key metabolites involved in the protective effects of AEE on vascular endothelial injury. However, these metabolites have not yet been further validated. Future studies will conduct functional validation of the enriched metabolites to fully elucidate the specific mechanisms by which AEE improves vascular endothelial injury and oxidative stress. This will provide a more robust theoretical foundation for the clinical translation of AEE.

## 4. Materials and Methods

### 4.1. Reagents

AEE (LOT:20190701, purity ≥ 99.6%) was chemically synthesized by the Lanzhou Institute of Husbandry and Pharmaceutical Sciences of CAAS (Lanzhou, China); Copper sulfate anhydrous, 99% (HY-Y1878C) was purchased MedChemExpress (Shanghai, China); 3% H_2_O_2_ solution (323381) was purchased Sigma-Aldrich (St. Louis, MS, USA); Ethanol absolute (10009259) was purchased Sinopharm Chemical Reagent Co., Ltd. (Shanghai, China); Fetal Bovine Serum (AUS-01E-02) was purchased Cell-Box (Changsha, China); DMEM (10565018) RPMI-1640 (11875093), 0.25% trypsin (25200072) was purchased Gibco (Grand Island, NE, USA); Cell Counting Kit-8 (C6050) was purchased NCM Biotech (Suzhou, China); Total Superoxide Dismutase Assay Kit with WST-8 (S0101M) was purchased Beyotime (Shanghai, China); Reduced Glutathione (GSH) Content Assay Kit (BC1175) was purchased Solarbio, (Beijing, China); Bovine Catalase (CAT) ELISA Kit (JL16435), Human Catalase (CAT) ELISA Kit (JL14741), Mouse Catalase (CAT) ELISA Kit (JL18163), Human Nuclear factor erythroid 2-related factor 2 (Nrf2) ELISA Kit (JL18367), Mouse Nuclear factor erythroid 2-related factor 2 (Nrf2) ELISA Kit (JL18277), Human Interleukin 6 (IL-6) ELISA Kit (JL14113), Mouse Interleukin 6 (IL-6) ELISA Kit (JL20268), Bovine Interleukin 6 (IL-6) ELISA Kit (JL22488), General 4-Hydroxynonenal (4-HNE) ELISA Kit (JL46304), General Malondialdehyde (MDA) ELISA Kit (JL53632), was purchased Jianglai Biotechnology (Shanghai, China); The MS-gradeacetonitrile was purchased Thermo Fisher Scientific (Waltham, MA, USA); The formic acid (98.0%, for LC-MS) was purchased Tokyo Chemical Industry (Shanghai, China); Deionized water (18.25 MΩ) was prepared with a Direct-Q^®^3 system (Millipore, Bedford, MA, USA).

### 4.2. Overview of Study Design

This study established injury models of BAEC, MAEC, and Huvecs using EtOH, H_2_O_2_, and CuSO_4_. The pretreatment concentration of AEE for protecting each VEC injury model was screened through cytomorphology, intracellular oxidative stress levels, and inflammation levels. Subsequently, metabolomic evaluation was performed to identify differential metabolites between the Model group and AEE group in difference VECs injury model, followed by pathway and functional enrichment analysis.

### 4.3. Enzyme-Linked Immunosorbent Assay

Levels of nuclear factor erythroid 2-related factor 2 (NRF2), CAT, IL-6, 4-hydroxynonena (4-HNE) and malondialdehyde (MDA) in cell were measured following the manufacturer’s instructions for the ELISA assay kit. Specifically, cells were seeded at a density of 1 × 10^5^ cells/mL in 6 well culture plates and subjected to corresponding treatments. After treatment, cells from each group were digested with trypsin and collected by centrifugation at 1000× *g* for 5 min. The collected cells were washed three times with pre-cooled PBS. To lyse the cells, 200 μL PBS was added, followed by three cycles of freezing–thawing in liquid nitrogen. The mixture was then centrifuged at 1500× *g* for 5 min at 4 °C, and the supernatant was collected for detection and protein concentration determination.

In this assay, 100 μL of sample or standard solutions were added to a pre-coated plate and incubated at 37 °C for 60 min. After discarding the liquid, 100 μL of biotin–antibody working solution was added and incubated again at 37 °C for 60 min, followed by three washes. Next, 100 μL of streptavidin-HRP working solution was added, incubated at 37 °C for 30 min, and washed five times. Then, 90 μL of tetramethylbenzidine substrate was added and incubated at 37 °C in the dark for 15 min, before adding 50 μL of stop solution to halt the reaction. Absorbance at 450 nm was measured, and concentrations of NRF2, CAT, IL-6, 4-HNE and MDA were calculated.

### 4.4. Cell Culture

BAECs were purchased from Guangzhou Genomed Biotechnology Co., Ltd. (Guangzhou, China). Huvecs and MAECs were purchased from Beina Biology (Beijing, China).

All cells were cultured in complete medium supplemented with 10% fetal bovine serum (FBS) and maintained in a humidified incubator at 37 °C with 5% CO_2_. The culture medium was replaced every 48 h. When cell confluency reached >90%, Cells were dissociated using trypsin. Cell passage numbers were controlled between 5 and 10 generations.

### 4.5. Cell Viability Assay

For the purpose of this study, stimuli linked to inducing oxidative stress in biological organisms—H_2_O_2_ [25], EtOH [5,6], and CuSO_4_ [7,8]—were selected as inducers to trigger damage and oxidative stress in VECs.

BAECs, HUVECs and MAEC in the logarithmic growth phase with healthy growth status were seeded into 96 well culture plates at a density of 1 × 10^5^ cells/mL. After 24 h, cells were treated with H_2_O_2-_, EtOH- or CuSO_4_-containing media at varying concentrations for different durations. Following drug treatment, the supernatant was aspirated, and 100 μL of CCK-8 detection working solution (prepared at a 1:9 ratio of CCK8 to culture medium) was added to each well. Plates were incubated at 37 °C for 2 h, and cell viability was calculated by measuring absorbance at 450 nm.

### 4.6. Cell Experiment Design

BAEC were seeded at 1 × 10^5^ cells/mL in culture flasks or plates and cultured for 48 h. After, injury models were established by treating cells with 500 μM H_2_O_2_ for 24 h, 8% EtOH for 3 h or 100 μM CuSO_4_ for 24 h.

Huvecs were seeded at 1 × 10^5^ cells/mL in culture flasks or plates and cultured for 48 h. After, injury models were established by treating cells 500 μM H_2_O_2_ for 24 h, 6% EtOH for 3 h or 100 μM CuSO_4_ for 24 h.

MAEC were seeded at 1 × 10^5^ cells/mL in culture flasks or plates and cultured for 48 h. After, injury models were established by treating cells with 500 μM H_2_O_2_ for 24 h, 5% EtOH for 3 h or 50 μM CuSO_4_ for 24 h.

Pretreatment with AEE (16, 32, 64 μM) was performed 24 h before injury model induction.

### 4.7. GSH Level Measurement

Cell samples were washed twice with PBS, and 400 μL of extraction solution was added according to the manufacturer’s instructions. Cell lysis was collected via frozen and thawed three times with liquid nitrogen, then centrifuged at 12,000× *g* for 4 min at 4 °C to obtain the supernatant for measurement. For GSH measurement, 20 μL of cell supernatant, 140 μL of Reagent 2, and 40 μL of Reagent 3 were combined and added to a 96-well plate. After thorough mixing, the mixture was incubated at room temperature for 2 min. Absorbance at 412 nm was then recorded to determine GSH concentrations in each sample.

### 4.8. SOD Activity Assay

Cell samples were washed twice with PBS, and 300 μL of extraction solution was added according to the manufacturer’s instructions. Cell lysis was centrifuged at 12,000× *g* for 4 min at 4 °C to obtain the supernatant for measurement. 20 μL of cell supernatant was added to a 96-well plate, followed by the addition of 140 μL of WST-8/enzyme working solution, and finally 20 μL of reaction initiation working solution. After mixing well, the plate was incubated at 37 °C for 30 min. Absorbance was measured at 450 nm, and the SOD activity of each sample was calculated.

### 4.9. Cell Metabonomic Analysis

Cell samples were washed twice with PBS, and 1mL 80% methanol was added, the samples were mixed and frozen at 80 °C for 10min. Cell lysis was collected via frozen and thawed three times with liquid nitrogen, then centrifuged at 14,000× *g* for 10 min at 4 °C to obtain the supernatant. Methanol was added to the supernatant (3:1, *v*/*v*), centrifuged at 14,000× *g* for 10 min at 4 C. 20 μL of supernatant from each sample were mixed to prepare a quality control sample. Then, the mixture was divided into aliquots with the same volume as other samples and prepared together. The supernatant was filtered through a 0.2 μm nylon mesh into sample vials.

Chromatographic separation was performed on an Agilent ZORBAX Eclipse Plus C18 RRHD analytical column (dimensions: 2.1 × 150 mm, particle size: 1.8 μm) using an Agilent 1290 Ultra Performance Liquid Chromatography (UPLC) system. The column was maintained at a constant temperature of 35 °C, with a 5 min equilibration period prior to sample injection. Elution was conducted at a flow rate of 0.25 mL/min, utilizing a binary mobile phase system consisting of (A) 0.1% (*v*/*v*) formic acid in ultrapure water and (B) 0.1% (*v*/*v*) formic acid in acetonitrile. The gradient elution program for cell samples was optimized as follows: 0–2 min at 5% B; 2–8 min ramping to 25% B; 8–20 min increasing to 95% B; 20–22 min maintaining 95% B; 22–23 min returning to 5% B; and 23–25 min holding at 5% B to re-equilibrate the column.

Mass spectrometric data were acquired using an Agilent 6530 Q-TOF mass spectrometer (Agilent Technologies, Santa Clara, CA, USA) equipped with an electrospray ionization (ESI) source, operating in both positive (ESI+) and negative (ESI-) ionization modes. Key ion source parameters were configured as follows: drying nitrogen gas was delivered at a flow rate of 10 L/min with a temperature of 350 °C; capillary voltages were set to 4.0 kV for ESI+ and 3.5 kV for ESI-; fragment or voltage was 135 V; skimmer voltage was 65 V; nebulizer pressure was 45 psig; and data were acquired at a rate of 1 spectrum per second. Mass data were collected across a range of 50–1000 *m*/*z* in centroid mode for subsequent analysis.

Raw data preprocessing was conducted using MS-DIAL 4.38 software to generate a high-quality feature table that included *m*/*z* values, retention times (RT), and peak areas. Subsequently, the processed data were subjected to multivariate statistical analysis via SIMCA 14.1 software, employing orthogonal partial least squares-discriminant analysis (OPLS-DA). Metabolites meeting the criteria of variable importance in projection (VIP) > 1, fold change (FC) < 0.83 or >1.20, and *p* < 0.05 were identified as potential differential metabolites between AEE group and Model group. Final confirmation of the differential metabolites of interest was achieved through spectral library matching in the Human Metabolome Database (http://www.hmdb.ca/ accessed on 20 July 2025). Based on the characterized differential metabolites, MetaboAnalyst 6.0 (http://www.metaboanalyst.ca/ accessed on 20 July 2025) was employed to conduct analysis of associated metabolic pathways.

### 4.10. Statistical Analysis

Data used for statistical analysis were subjected to the Shapiro–Wilk test (for normality assessment) and Brown–Forsythe test (for homogeneity of variance assessment). Subsequently, differential analysis was performed using One-way ANOVA followed by Tukey’s multiple comparisons test, implemented via GraphPad Prism 10.1.2 software. Data are represented as mean ± SD. The significance level of *p* < 0.05 was considered statistically significant.

## Figures and Tables

**Table 1:** Potential biomarkers in EtOH-induced cell injury model.

Inducer	Cell	Metabolites	Formula	SM	RT	*m*/*z*	VIP	FC (AEE/M)
EtOH	BAEC	Uridine	C_9_H_12_N_2_O_6_	EST+	1.68	245.0780	1.3543	10.0367 **
		2-(S-Glutathionyl) acetyl glutathione	C_22_H_34_N_6_O_13_S_2_	EST−	17.084	653.1437	1.8796	7.1396 *
		4′-Phosphopantothenoylcysteine	C_12_H_23_N_2_O_9_PS	EST−	15.316	401.0808	1.8739	2.1642 **
		4-Hydroxybenzoic acid	C_7_H_6_O_3_	EST−	18.097	137.0226	2.4293	0.0008 ***
	MAEC	Glutamic acid	C_5_H_9_NO_4_	EST+	2.478	148.0577	1.2428	1.3132 **
		Glutathione	C_10_H_17_N_3_O_6_S	EST+	2.528	308.0857	1.5946	27.7886 *
		Adenosine monophosphate	C_10_H_14_N_5_O_7_P	EST+	2.715	348.0651	1.1843	0.4544 *
		Phytosphingosine	C_18_H_39_NO_3_	EST+	17.271	318.2955	1.5514	27.4347 *
		4′-Phosphopantothenoylcysteine	C_12_H_23_N_2_O_9_PS	EST−	17.503	401.0853	1.8519	15.0864 ***
	Huvecs	Homovanillin	C_9_H_10_O_3_	EST+	2.75	167.0718	1.1007	21.6488 *
		Tetrahydrocorticosterone	C_21_H_34_O_4_	EST+	3.857	351.2467	1.1185	78.0069 *
		1,2-Dihydronaphthalene-1,2-diol	C_10_H_10_O_2_	EST+	17.979	163.0768	1.5327	1.2113 **
		Phytosphingosine	C_18_H_39_NO_3_	EST+	18.176	318.2978	1.242	3.8590 *
		4′-Phosphopantothenoylcysteine	C_12_H_23_N_2_O_9_PS	EST−	18.517	401.0848	1.0302	9.8905 *
		Glutathione	C_10_H_17_N_3_O_6_S	EST−	6.167	306.0768	1.715	3.0495 **

SM, scan mode; EST+, metabolites identified in positive mode; EST−, metabolites identified in negative mode; RT, retention time; *m*/*z*, mass-to-charge ratio; VIP, variable importance in projection; FC, fold change. Metabolites identified in both positive and negative modes; * *p* < 0.05, ** *p* < 0.01, and *** *p* < 0.001. AEE/M: AEE vs. Model.

**Table 2:** Potential biomarkers in H_2_O_2_-induced cell injury model.

Inducer	Cell	Metabolites	Formula	SM	RT	*m*/*z*	VIP	FC (AEE/M)
H_2_O_2_	BAEC	Proline	C_5_H_9_NO_2_	EST+	2.322	116.0694	1.0932	19.3354 *
		L-Valine	C_5_H_11_NO_2_	EST+	2.458	118.0844	1.1375	13.3350 *
		Glutathione	C_10_H_17_N_3_O_6_S	EST+	3.595	308.0879	1.0257	3.9698 *
		Phenylalanine	C_9_H_11_NO_2_	EST+	7.186	166.083	1.1525	3.1309 **
		Sphinganine	C_18_H_39_NO_2_	EST+	15.967	302.3035	1.1289	11.1349 *
		Uridine diphosphate glucose	C_15_H_24_N_2_O_17_P_2_	EST−	3.739	565.0413	1.3973	1.6738 **
	MAEC	all-trans-Retinoic acid	C_20_H_28_O_2_	EST+	16.648	301.2169	1.0836	28.7524 *
		4′-Phosphopantothenoylcysteine	C_12_H_23_N_2_O_9_PS	EST−	16.728	401.0863	1.0771	1.6261 *
		1-Methylxanthine	C_6_H_6_N_4_O_2_	EST−	19.145	165.0392	1.2266	1.2591 *
	Huvecs	Choline	C_5_H_14_NO	EST+	2.135	104.1063	1.5907	6.0157 *
		Sphingosine	C_18_H_37_NO_2_	EST+	16.601	300.2878	1.8422	0.7391 **
		Eicosapentaenoic acid	C_20_H_30_O_2_	EST+	16.838	303.2281	2.0591	13.4526 **
		Isocitric acid	C_6_H_8_O_7_	EST−	11.081	191.0169	1.2282	16.0554 *
		Arachidonic acid	C_20_H_32_O_2_	EST−	19.186	303.2271	1.6681	0.7465 *
		Palmitic acid	C_16_H_32_O_2_	EST−	19.708	255.2294	1.7891	0.7148 *

SM, scan mode; EST+, metabolites identified in positive mode; EST−, metabolites identified in negative mode; RT, retention time; *m*/*z*, mass-to-charge ratio; VIP, variable importance in projection; FC, fold change. Metabolites identified in both positive and negative modes; * *p* < 0.05, ** *p* < 0.01. AEE/M: AEE vs. Model.

**Table 3:** Potential biomarkers in CuSO_4_-induced cell injury model.

Inducer	Cell	Metabolites	Formula	SM	RT	*m*/*z*	VIP	FC (AEE/M)
CuSO_4_	BAEC	LysoPC(16:0/0:0)	C_24_H_50_NO_7_P	EST+	19.8	496.3367	1.7987	0.7937 *
		Phytosphingosine	C_18_H_39_NO_3_	EST+	3.122	318.2987	1.752	3.0271 **
		3-Dehydrosphinganine	C_18_H_37_NO_2_	EST+	16.576	300.2889	1.001	0.8109 *
		Phenylethylamine	C_8_H_11_N	EST+	0.102	122.0952	1.3499	7.0647 **
		4-Hydroxybenzoic acid	C_7_H_6_O_3_	EST−	18.996	137.0237	1.6976	0.0541 ***
		Palmitic acid	C_16_H_32_O_2_	EST−	19.707	255.2305	1.7083	0.7595 **
	MAEC	Choline	C_5_H_14_NO	EST+	2.61	104.1059	1.8884	6.6366 **
		4′-Phosphopantothenoylcysteine	C_12_H_23_N_2_O_9_PS	EST−	18.57	401.0835	1.7491	11.6252 ***
		Uridine 5′-monophosphate	C_9_H_13_N_2_O_9_P	EST−	5.383	323.0242	1.0491	0.8200 **
		6-Methylmercaptopurine	C_6_H_6_N_4_S	EST−	17.848	165.0189	1.4829	1.2122 *
	Huvecs	Choline	C_5_H_14_NO	EST+	2.102	104.1061	1.3179	1.3354 *
		Hypoxanthine	C_5_H_4_N_4_O	EST+	2.434	137.0453	1.6656	0.1555 *
		Homovanillin	C_9_H_10_O_3_	EST−	15.6	165.0539	1.4198	5.8969 *

SM, scan mode; EST+, metabolites identified in positive mode; EST−, metabolites identified in negative mode; RT, retention time; *m*/*z*, mass-to-charge ratio; VIP, variable importance in projection; FC, fold change. Metabolites identified in both positive and negative modes; * *p* < 0.05, ** *p* < 0.01, and *** *p* < 0.001. AEE/M: AEE vs. Model.

## Data Availability

The data that support the findings of this study are available from the corresponding author upon reasonable request.

## Supplementary Materials

The following supporting information can be downloaded at: https://www.mdpi.com/article/10.3390/ijms27020877/s1.
