# Supplementary material for: Multicellular Model Reveals the Mechanism of AEE Alleviating Vascular Endothelial Cell Injury via Anti-Inflammatory and Antioxidant Effects
Source: Int J Mol Sci. 2026 Jan 15;27(2):877. doi: 10.3390/ijms27020877 (PMC12841016; doi:10.3390/ijms27020877)
Supplement: Supplementary file 1 [file ijms-27-00877-s001.zip › ijms-4016990-supplementary.pdf]

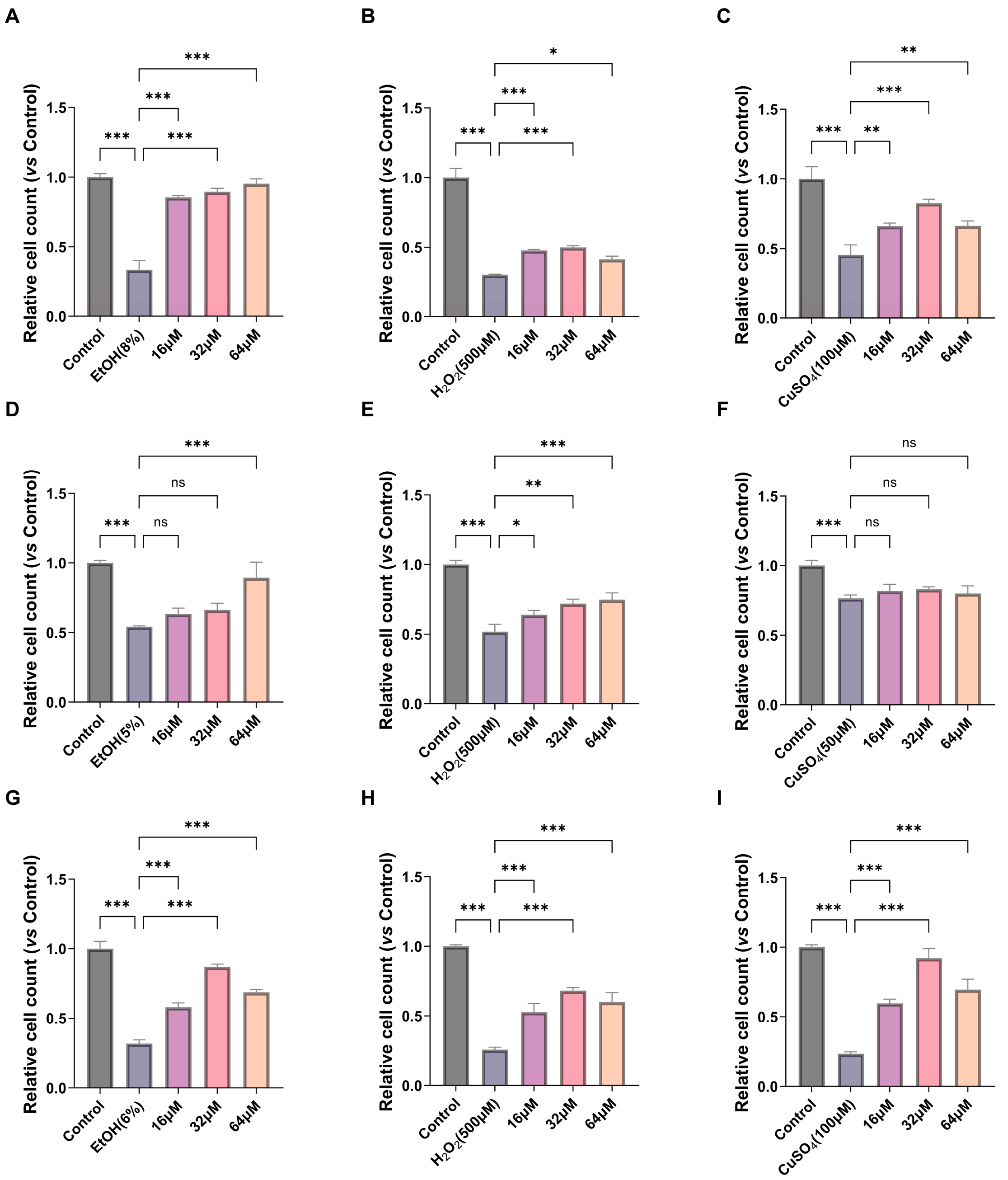

Figure S1 Cell Number of the Effects of AEE on Vascular Endothelial Injury Models. (A-C) Semi-quantitative analysis of the effects of AEE pretreatment on changes in cell number in BAEC injury models induced by EtOH, H_2O_2, and $CuSO_4$ (n = 3). (D-F) Semi-quantitative analysis of the effects of AEE pretreatment on changes in cell number in MAEC injury models induced by EtOH, H_2O_2, and $CuSO_4$ (n = 3). (G-I) Semi-

quantitative analysis of the effects of AEE pretreatment on changes in cell number in Huvecs injury models induced by EtOH, H_2O_2, and $CuSO_4$ (n = 3). Data are represented as mean ± SD. Comparison among groups: ns $P > 0.05$, *$P < 0.05$, **$P < 0.01$, ***$P < 0.001$.
